# Mechanisms of acute neurovascular protection with AT1 blockade after stroke: Effect of prestroke hypertension

**DOI:** 10.1371/journal.pone.0178867

**Published:** 2017-06-22

**Authors:** Ahmed Alhusban, Anna Kozak, Bindu Pillai, Heba Ahmed, Mohammed A. Sayed, Maribeth H. Johnson, Tauheed Ishrat, Adviye Ergul, Susan C. Fagan

**Affiliations:** 1 Program in Clinical and Experimental Therapeutics- Charlie Norwood VA Medical Center and College of Pharmacy, University of Georgia, Augusta, Georgia, United States of America; 2 College of Pharmacy, Jordan University of Science and Technology, Irbid, Jordan; 3 Departments of Biostatistics, Medical College of Georgia, Augusta University, Augusta, Georgia, Unites States of America; 4 Department of Physiology, Medical College of Georgia, Augusta University, Augusta, Georgia, United States of America; 5 Department of Neurology, Medical College of Georgia, Augusta University, Augusta, Georgia, United States of America; Fraunhofer Research Institution of Marine Biotechnology, GERMANY

## Abstract

Stroke is a leading cause of adult disability worldwide. Improving stroke outcome requires an orchestrated interplay that involves up regulation of pro-survival pathways and a concomitant suppression of pro-apoptotic mediators. In this investigation, we assessed the involvement of eNOS in the AT1 blocker-mediated protective and pro-recovery effects in animals with hypertension. We also evaluated the effect of acute eNOS inhibition in hypertensive animals. To achieve these goals, spontaneously hypertensive rats (SHR) were implanted with blood pressure transmitters, and randomized to receive either an eNOS inhibitor (L-NIO) or saline one hour before cerebral ischemia induction. After 3 hours of ischemia, animals were further randomized to receive either candesartan or saline at the time of reperfusion and sacrificed either 24 hours or 7 days later. Candesartan induced an early protective effect that was independent of eNOS inhibition (50% improvement in motor function). However, the protective effect of candesartan was associated with about five fold up regulation of BDNF expression and about three fold reduction in ER stress markers, in an eNOS dependent manner. The early benefit of a single dose of candesartan, present at 24 hours after stroke, was diminished at 7 days, perhaps due to a failure to induce an angiogenic response in these hypertensive animals. In conclusion, our findings demonstrate an early prorecovery effect of candesartan at both functional and molecular levels. Candesartan induced prorecovery signaling was mediated through eNOS. This effect was not maintained at 7 days after experimental ischemia.

## Introduction

Data from our lab and others have demonstrated a robust neurovascular protective and pro-recovery effect of angiotensin II receptor blockers (ARBs) after stroke [1–11]. The early neurovascular protective effect is likely mediated through a number of mechanisms including cerebral blood flow improvement [5], oxidative stress and endothelial dysfunction amelioration [12–14], and upregulation of endothelial nitric oxide (eNOS) expression [15]. The latter two mechanisms are of particular interest, since they directly address the ravaging effects of chronic angiotensin II Type 1 receptor activation, seen in hypertension, on the cerebral circulation [16]. We have proposed that the pro-recovery effect of ARBs after stroke in normotensive animals is due to enhanced growth factor expression and subsequent reparative angiogenesis [2, 3]. Since hypertension is exceedingly common in stroke patients, it is highly important to study the interaction between ARBs and hypertension and how this affects both early injury and later stroke outcome.

We previously demonstrated the ability of AT1 blockers to increase the expression of both vascular endothelial growth factor (VEGF) and brain derived neurotrophic factor (BDNF) at 24 hours after stroke in normotensive animals, and this was associated with increased vascular density and improved outcome at 7 days [3]. Furthermore, we demonstrated the ability of subhypotensive doses of candesartan to increase the expression of BDNF at 24 hours after experimental ischemia in normotensive animals and this was shown to be reperfusion dependent [17]. The increase in growth factors was also demonstrated in normotensive and hypertensive animals not exposed to cerebral ischemia [18]. BDNF exerts it’s pro-recovery effect via the mature form, which is generated by the conversion of proBDNF to mature BDNF. It is still unknown whether ARBs are involved in the de novo expression of proBDNF or the observed increase in mature BDNF levels results from enhanced conversion of the pro-form into the mature form. In addition, it is still unknown whether the same effect will be maintained after stroke in hypertensive animals. Endothelial NOS has been shown to be essential for the expression of brain derived neurotrophic factor (BDNF) [19]. Chen et al., induced stroke in eNOS knockout mice and assessed the extent of neurogenesis and BDNF expression. Their findings demonstrated that eNOS knockout animals have lower levels of BDNF and neurogenesis. Additionally, BDNF has been shown to be involved in recovery after stroke [18, 20–22]. It is still unknown, whether eNOS is involved in ARB-induced BDNF expression in hypertensive animals after stroke. Furthermore, it is unknown whether eNOS is involved in the conversion of proBDNF to mature BDNF.

Angiogenesis has been shown to be involved in recovery after a variety of CNS ischemia insults [19, 23]. Data from our lab showed a sustained prorecovery effect of a single dose of candesartan after 7 days of experimental ischemia induction in normotensive animals [2]. This effect was associated with a proangiogenic state and increased vascular density [2]. It is still unknown whether the same effect would be observed in hypertensive animals after experimental ischemia.

The purpose of this investigation was to assess: 1) The importance of eNOS in the neurovascular protective effects of AT1 receptor antagonism, 2) The interaction between AT1 blockade, eNOS and neurotrophin signaling and development of endoplasmic reticulum (ER) stress after stroke and, 3) To determine the effects of early AT1 blockade on 7 day neurobehavioral outcomes in hypertensive rats.

## Materials and methods

### Animals

**Fig 1:**
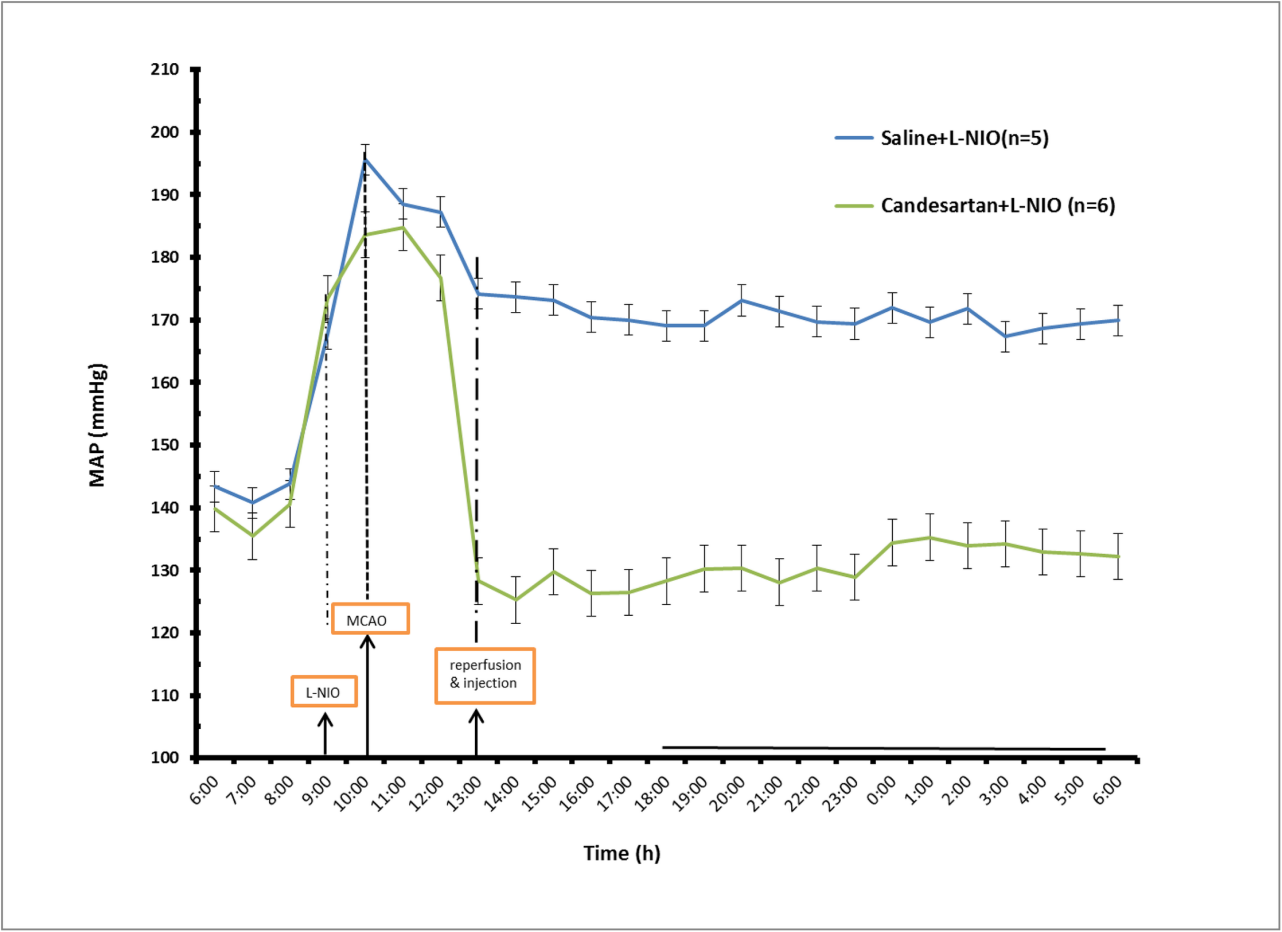
Acute eNOS inhibition did not alter early AT1 blocker treatment induced reduction of blood pressure after the induction of cerebral ischemia. A schematic diagram showing the experimental design (A). Blood pressure telemetry showing the hypotensive effect of Candesartan following cerebral ischemia. This effect was not altered with acute eNOS inhibition before the induction of cerebral ischemia. Data presented as mean±SEM; n = 5–6 animals per group.

The experimental protocol was approved by the Institutional Animal Care and Use Committee (IACUC) of the Charlie Norwood Veterans Affairs Medical Center (09-04-008). Eight week old, male spontaneously hypertensive rats (SHRs) (220–250 g; n = 6–8 per group) were purchased from Charles River laboratory and implanted with blood pressure transmitters as previously reported [2, 24]. After recovery, blood pressure was monitored throughout the duration of the experiment. Animals were randomized to receive either a single dose of an eNOS inhibitor, N5- (1- iminoethyl)—L- ornithine, hydrochloride (L-NIO) (Cayman chemical; Ann Arbor, MI) or saline one hour before inducing ischemia. For the 24 hour study, cerebral ischemia was induced through temporary middle cerebral artery occlusion (tMCAO) for 3 hours and for the 7 day study, 60 minute tMCAO was employed [1]. We chose 60 minute MCAO to reduce the degree of CNS damage so that the animals would survive for the full 7 day follow-up period [25, 26]. At 3 hours after the onset of tMCAO, animals were further randomized to receive either saline or 1 mg/kg candesartan IV (Astra-Zeneca). This dose was previously shown to improve functional outcome at 24 hours in SHRs [24]. Animals were then subjected to stroke and followed up for 24 hours or 7 days (Fig 1A). Prior to sacrifice, animals were deeply anesthestized and perfused with ice-cold phosphate buffered saline. Brains were harvested, and hemispheres separated, and flash frozen in liquid nitrogen.

### Animal care

Animals were anesthetized with isoflurane inhalation 1.5–3% and received buprenorphine 0.05 mg/kg sc before reperfusion and at 12 hours after MCAO, if necessary. In addition, animals received a 3 mm ribbon of lidocaine 5% ointment to their surgical wound and were monitored for signs of distress every 30 minutes for 3 hours and twice daily after that until sacrifice. The animals were weighed daily.

### Behavioral and functional outcome analysis

Functional outcome was evaluated in a blinded manner at 24 hours, 4, and 7 days after stroke, using the Bederson score, beam walk and paw grasp tests, as reported previously [2].

### Western blotting

Brains were homogenized and processed for western blotting as previously described [27]. Thirty μg of proteins were loaded in each lane and separated followed by transfer to nitrocellulose membranes. The membranes were blocked using 5% non-fat milk in TBST (1% tween 20 in tris buffered saline) and probed with the following antibodies antiBDNF (1:250; abcam; Cambridge, MA), TrkB (1:500, abcam; Cambridge, MA), p75NTR (1:1000, Millipore; Billerica, MA), Nogo-A (1:1000, Millipore; Billerica, MA), CHOP (1:1000) and nNOS (1:1000, Cell Signaling; Danvers, MA), ATF6 (1:500), pJNK (1:1000) and JNK (1:1000, Santa Cruz biotechnologies; Santa Cruz, CA). Expression was assessed by quantification of optical density of respective bands normalized to actin using NIH-image J software.

### Hemoglobin assay

Microscopic bleeding was quantified using a colorimetric hemoglobin detection assay (QuantiChrom Hemoglobin Assay Kit, BioAssay Systems, Haywood, CA). First, TTC-stained brain samples were homogenized in a 10% glycerol-Tris-buffered saline solution containing Tween 20. Samples were prepared and read at 562 nm using a standard microplate reader, and the hemoglobin concentration was calculated according to the manufacturer's instructions. Bleeding was expressed as the excess hemoglobin in the ischemic hemisphere.

### Nitrosative stress

Nitrosative stress was quantified using slot blotting. Briefly, 30 μg of proteins of each sample were loaded in each cell of the slot blot apparatus. Vacuum was applied to transfer the proteins to the nitrocellulose paper. The membranes were blocked using 5% non-fat milk in TBST (1% tween 20 in tris buffered saline) for 1 hour. Membranes were probed with anti-Nitro-tyrosine antibody (Cayman chemical; Ann Arbor, MI) Nitro-tyrosine levels were quantified by measuring the optic density of the bands using image J software.

### Vascular density

At day 7 after stroke, rats were given an overdose of ketamine/xylazine and then transcardially perfused with cold saline followed by 10% buffer-formalin via the ascending aorta. The brains were removed and postfixed in 10% buffer-formalin (Fischer Scientific) for 48 h and then stored at 4°C in a solution of 30% sucrose–saline for 2 days. The brains were embedded in OCT and sectioned coronally in 12-μm-thick slices starting from the frontal pole at an interval of 2 mm. Primary antibodies were incubated overnight at 4°C at the following dilutions: rabbit anti-laminin (1/200; Dako Cytomation, Carpinteria, California, USA). After washing, slides were incubated with fluorescent secondary antibodies, cover-slipped with Vectashield mounting medium (Vector Laboratories, Burlingame, California, USA) and viewed using ZeisAxio Observer.Z1 fluorescent microscope. Negative controls were prepared by omitting the primary antibodies. Laminin-stained vascular profiles (including arterioles and venules) were quantified using ImageJ software (NIH) in 3 different fields per section digitized from the ischemic border zone using a 10X objective lens. An average of the 3 fields of interest (FOI) per animal was calculated and plotted.

### Statistical analysis

Data were assessed for normality and a log transformation was used when a distribution was skewed or when the variance increased with the mean. Transformed variables included hemoglobin excess, BDNF, pro-BDNF, TrkB, p75NTR, Nogo A, and ATF6. Area under the curve (AUC) for blood pressure was calculated for the three hours prior to L-NIO injection (PRE), the three hours of temporary middle cerebral artery occlusion (tMCAO), and the seventeen hours post reperfusion and injection (POST). The remaining variables were analyzed using a 2 Candesartan (no vs. yes) by 2 L-NIO (no vs. yes) ANOVA. Interactions were included in all analyses to assess a differential effect of candesartan in the presence of L-NIO. Paw grasp and beam walk severity score proportions were analyzed for group differences using Fisher’s exact test. SAS® version 9.3 (SAS Institute, Inc., Cary, NC) was used for all analyses. Statistical significance was determined at alpha = 0.05.

## Results

### Acute eNOS inhibition does not affect the hypotensive effect of candesartan

Endothelial nitric oxide synthase was acutely inhibited using L-NIO before induction of experimental ischemia with continuous blood pressure monitoring. Acute eNOS inhibition did not affect the hypotensive effect of candesartan (Fig 1B).

### Acute eNOS inhibition does not alter the effect of candesartan on hemorrhagic transformation

**Fig 2:**
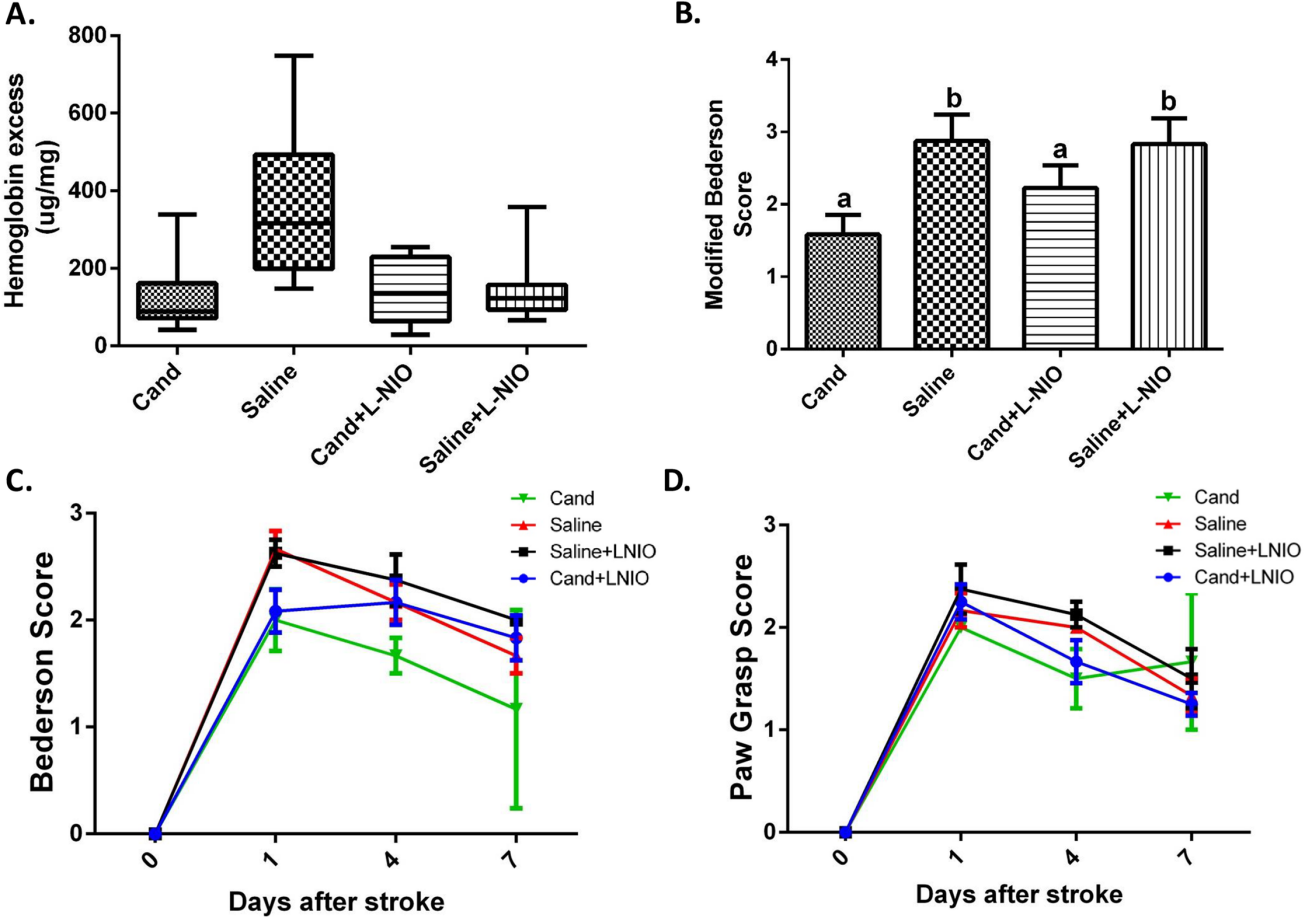
Early AT1 blockade improved 24-hour functional outcome measures while not affecting hemorrhagic transformation in hypertensive animals. Neither candesartan nor L-NIO had an effect on the development of hemorrhagic transformation (A). Candesartan improved neurological outcome after stroke, as assessed by modified Bederson, in an eNOS independent manner (B). After 7 days, there was a significant effect of time (p<0.01) in all measures of neurobehavioral outcome, demonstrating a gradual recovery from the initial deficits in all treatment groups. The candesartan-treated group (without eNOS inhibition) had better Bederson (2C) and Paw grasp (2D) scores than the other groups, but neither reached statistical significance. a, b: Pairs of means with different letters are significantly different from each other. Data presented as mean±SEM; n = 6–8 per group. Statistical analysis was done as a 2×2 factorial analysis. For clarity, animals that received saline injections before the induction of tMCAO are identified by the treatment they received after tMCAO only.

We assessed whether acute eNOS inhibition would alter the effect of candesartan on hemorrhagic transformation. Acute eNOS inhibition did not alter the degree of hemorrhagic transformation, alone or in combination with candesartan (Fig 2A).

### Acute eNOS inhibition did not affect candesartan induced neurobehavioral improved outcome

Acute eNOS inhibition did not alter candesartan induced improvement in motor function assessed 24 hours after cerebral ischemia (Fig 2B). In the 7 day study, there was a significant effect of time (p<0.01) in all measures of neurobehavioral outcome, supporting a gradual recovery from the initial deficits. The candesartan-treated group (without eNOS inhibition) had better Bederson (Fig 2C) and Paw grasp (Fig 2D) scores than the other groups, but neither reached statistical significance.

### eNOS mediates AT1 blockade induced BDNF expression

**Fig 3:**
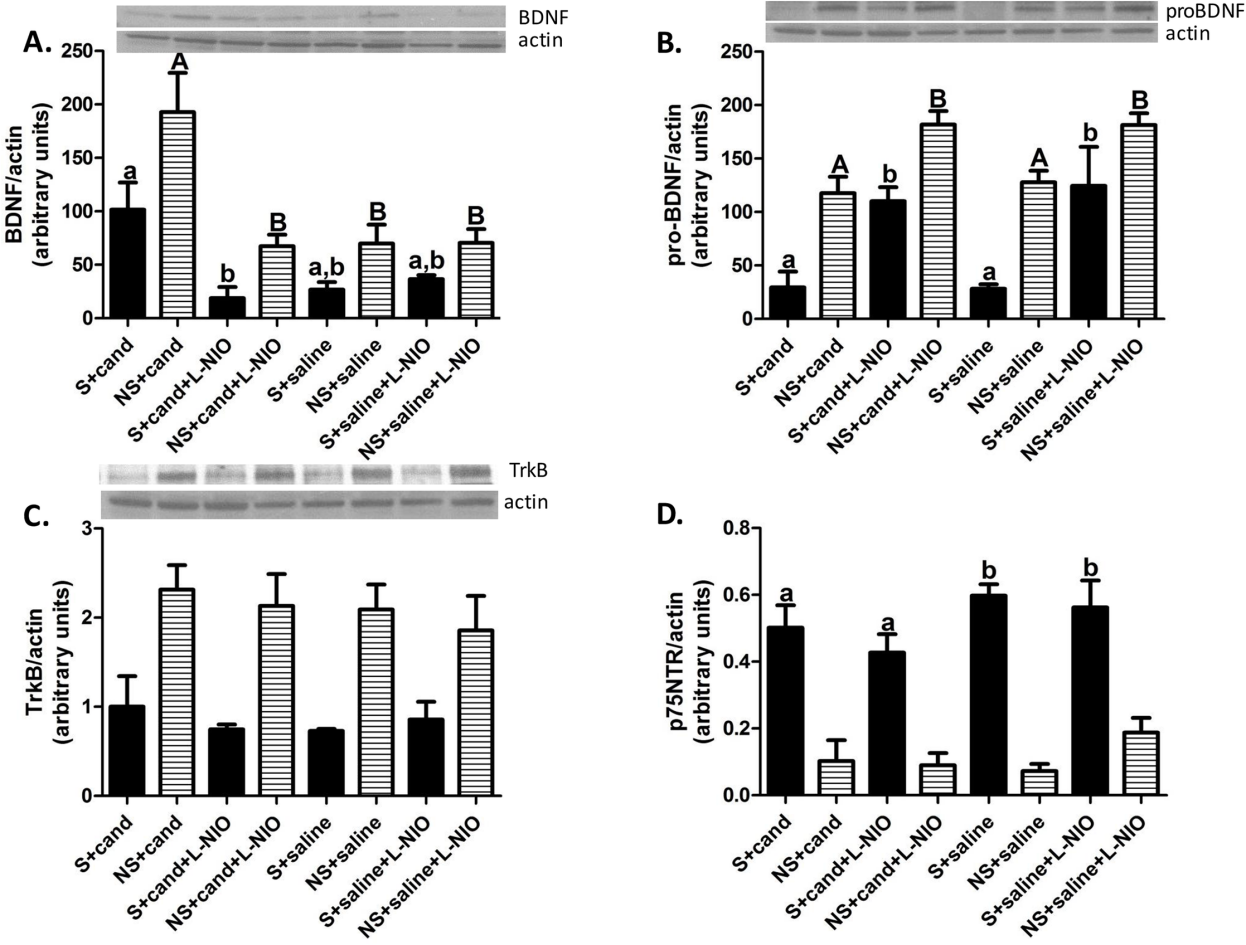
Early AT1 blockade enhanced the prosurvival signaling of BDNF/TrkB in an eNOS dependent manner. Candesartan increased BDNF expression in an eNOS dependent manner (F = 8.96, df = 3; p = 0.035) (A). eNOS inhibition increased proBDNF expression in both hemispheres (ipsilateral F = 5.5, df = 3; p = 0.018; contralateral = 7.27, df = 3; p = 0.007)(B). eNOS inhibition increased p75NTR in the ipsilateral hemisphere (p value for interaction = 0.038)(D). TrkB expression was not altered by any of the used interventions (C). Data presented as mean±SEM. Solid columns represent ipsilateral hemisphere, striped columns represent contralateral hemisphere. S refers to the ipsilateral hemisphere; NS refers to the contralateral hemisphere. a, b or A, B Pairs of means with different letters and/or capitalization are significantly different from each other. n = 4 animals per group.

At 24 hours after cerebral ischemia, candesartan significantly increased BDNF expression in the contralesional hemisphere when compared to saline treated animals (Fig 3A). Acute eNOS inhibition ablated the candesartan-induced increase in BDNF expression (Fig 3A).

### eNOS is involved in proBDNF processing

Acute eNOS inhibition increased proBDNF expression in saline treated animals (Fig 3B). Consistent with findings in saline-treated animals, acute inhibition of eNOS significantly increased proBDNF levels in the ipsilateral hemisphere of candesartan-treated animals (Fig 3B). Combined together, these findings suggest a potential role of eNOS in the conversion of proBDNF into mature form. Additionally, this highlights a possible eNOS-dependent effect of candesartan on the conversion of proBDNF into the mature form.

### AT1 blockade does not affect TrkB expression in hypertensive animals

Candesartan did not alter the expression of TrkB in SHRs (Fig 3C). Additionally, acute eNOS inhibition did not alter TrkB expression (Fig 3C).

#### Early AT1 blockade reduced p75NTR expression in the ipsilateral hemisphere

**I**nduction of cerebral ischemia resulted in a robust increase of p75NTR expression in the ipsilateral hemisphere (Fig 3D). Interestingly, candesartan treatment was associated with reduced p75NTR expression in the ipsilateral hemisphere of SHRs (Fig 3D). This effect was not affected by eNOS inhibition.

### Acute eNOS inhibition increases the expression of nNOS in the ipsilateral hemisphere after cerebral ischemia

**Fig 4:**
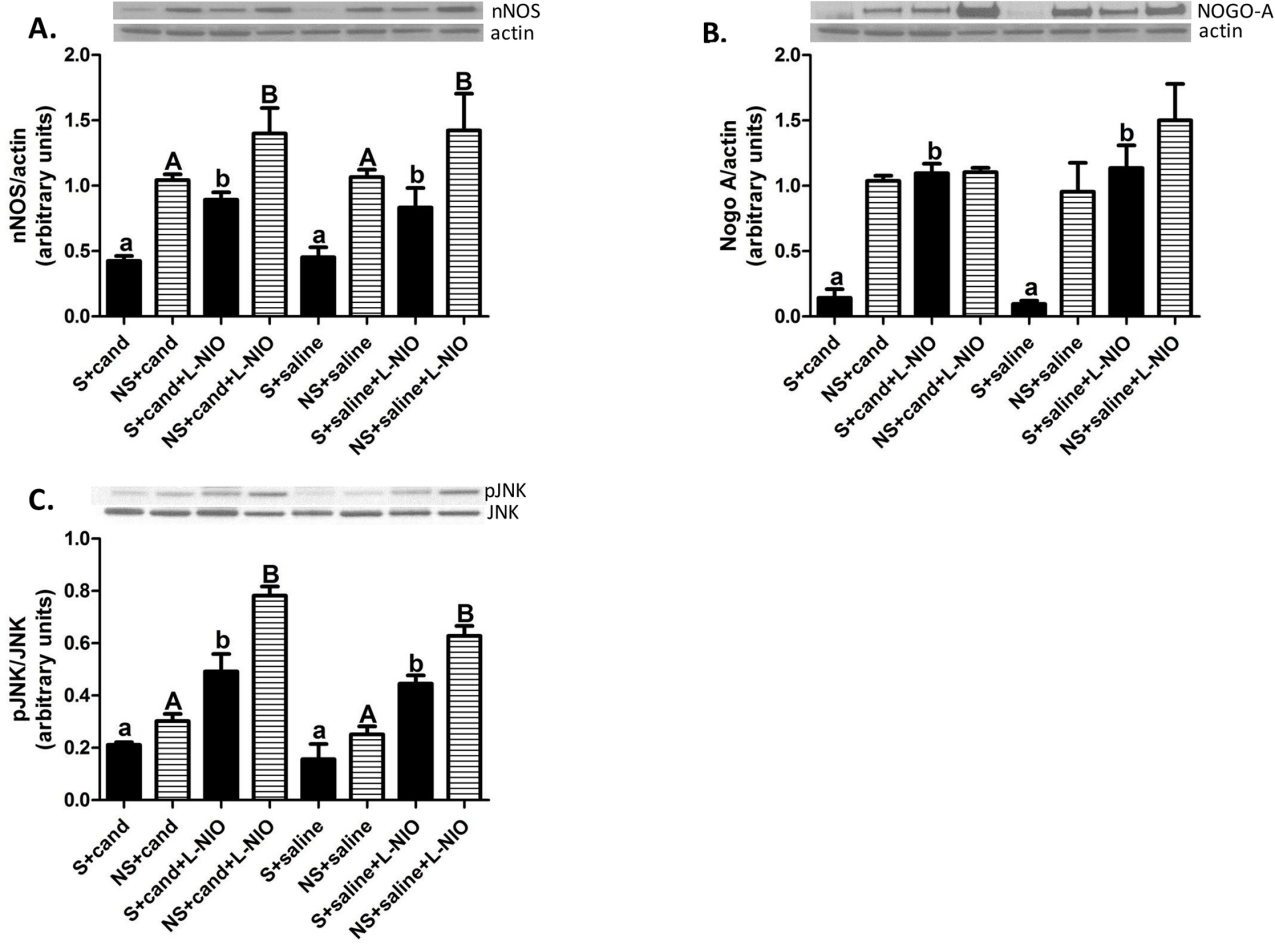
eNOS inhibition induces the expression and activity of anti-recovery mechanisms after cerebral ischemia. Acute L-NIO treatment increased nNOS (F = 7.44, df = 3; p = 0.0014) (A) and Nogo-A expression (B) in the ipsilateral hemisphere (F = 11.68 df = 3; p<0.0001) This increase was associated with a concomitant increase in JNK phosphorylation (F = 28.46, df = 3; p<0.0001) (C). Data presented as mean±SEM. Solid columns represent ipsilateral hemisphere, columns with stripes represent contralateral hemisphere. S refers to the ipsilateral hemisphere; NS refers to the contralateral hemisphere. a, b or A, B Pairs of means with different letters are significantly different from each other. n = 6–8 animals per group.

Our findings demonstrate a deleterious effect of acute eNOS inhibition on stroke outcome. Accordingly, we aimed at further explaining the molecular basis of eNOS inhibition mediated effects. eNOS inhibition induced a robust upregulation of nNOS expression in both hemispheres of candesartan and saline treated hypertensive animals (Fig 4A).

### Acute eNOS inhibition increased NOGO-A expression in the ipsilateral hemisphere after cerebral ischemia

Induction of cerebral ischemia decreased the level of NOGO-A in the ipsilateral hemisphere of SHRs as compared to contralesional hemisphere (Fig 4B). AT1 blockade did not have any appreciable effect on NOGO-A expression when compared to saline treated animals. Interestingly, acute eNOS inhibition upregulated the expression of NOGO-A in the ipsilateral hemisphere of both candesartan and saline treated SHRs after MCAO (Fig 4B). The expression of NOGO-A in the contralateral hemisphere was not altered by any of the applied treatments.

Consistent with changes in NOGO-A expression, L-NIO treatment significantly increased JNK phosphorylation in the ipsilateral hemispheres of both candesartan and saline treated animals (Fig 4C). Additionally, acute eNOS inhibition increased JNK phosphorylation in the contralesional hemisphere. AT1 blockade did not affect JNK phosphorylation. These findings suggest a causal link between eNOS inhibition and NOGO-A expression that is unrelated to AT1 blockade.

### AT1 blockade ameliorates ER stress and reduces UPR markers

**Fig 5:**
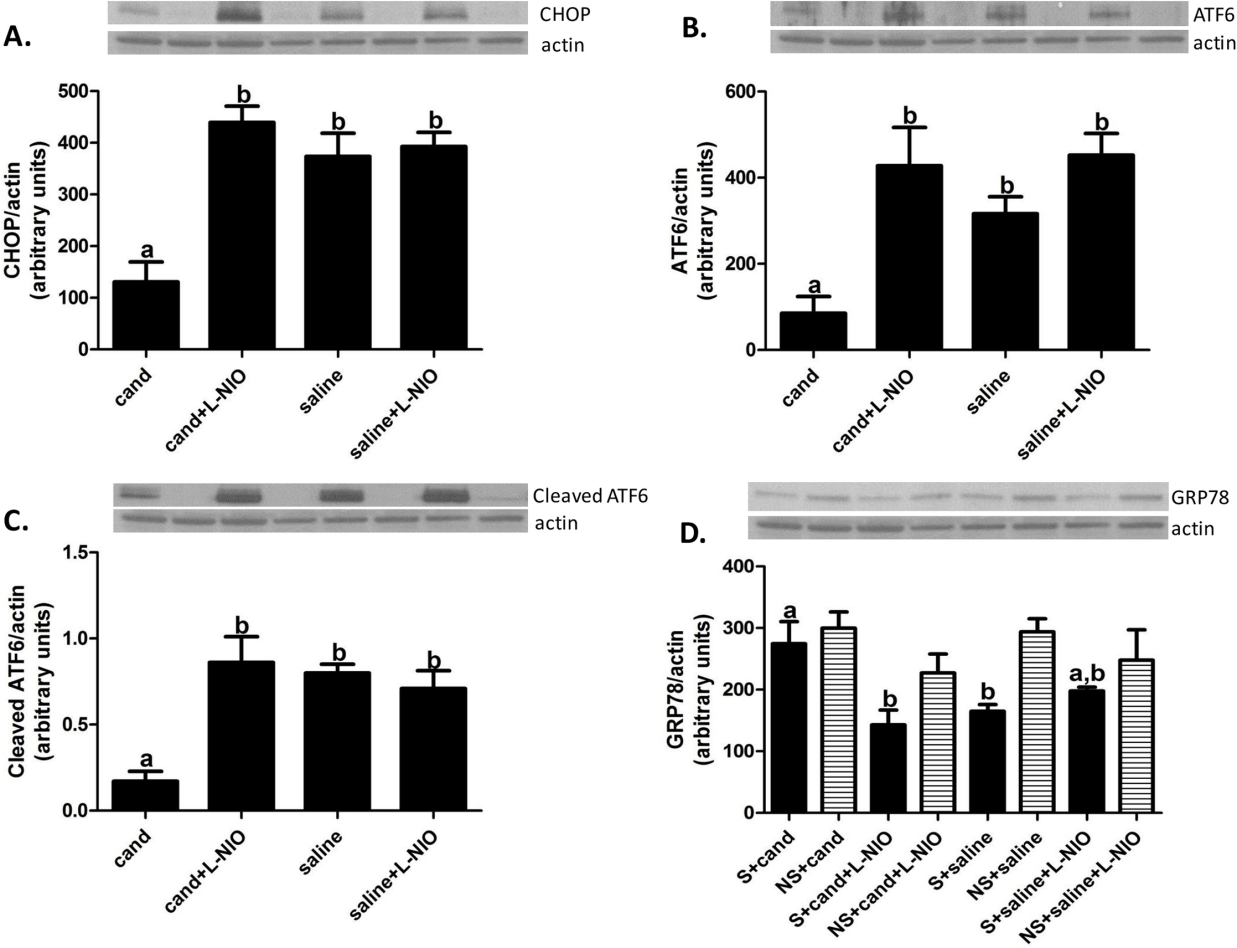
Early AT1 blockade ameliorates ischemia-induced increase in ER stress. Candesartan treatment at time of reperfusion reduced the expression of CHOP (F = 12.52, df = 3: p = 0.0007) (A), cleaved ATF6 (F = 7.88; df = 3; p = 0.0044) (C) and increased GRP78 expression in an eNOS dependent manner (F = 6.433, df = 3; p = 0.0089)(D). eNOS inhibition increased the expression of full length ATF6 (F = 8.264, df = 3; p = 0.0078) (B) Data presented as mean±SEM. Solid columns represent ipsilateral hemisphere, columns with stripes represent contralateral hemisphere. S refers to the ipsilateral hemisphere; NS refers to the contralateral hemisphere. a, b Pairs of means with different letters are significantly different from each other * n = 4 per group.

Candesartan administration counteracted ischemia- induced increase in CCAAT-enhancer-binding protein homologous protein(CHOP) levels (Fig 5A). Interestingly, candesartan reduced the cleavage of activating transcription factor 6 (ATF6) as measured by cleaved ATF6 in the brain (Fig 5C). Overexpression of GRP78 has been shown to reverse ER stress associated hypertension [28]. Candesartan significantly increased GRP78 expression in both hemispheres of SHRs (Fig 5D).

Acute eNOS inhibition consistently ablated AT1 blockade-mediated amelioration of ER stress in hypertensive animals after stroke (Fig 5A, 5B, 5C and 5D). These findings confirm previous reports on the involvement of eNOS in ER stress development in extracranial tissues and, for the first time, demonstrate this finding in the brain after stroke.

### Acute eNOS inhibition increases nitrosative stress in the contralesional hemisphere

**Fig 6:**
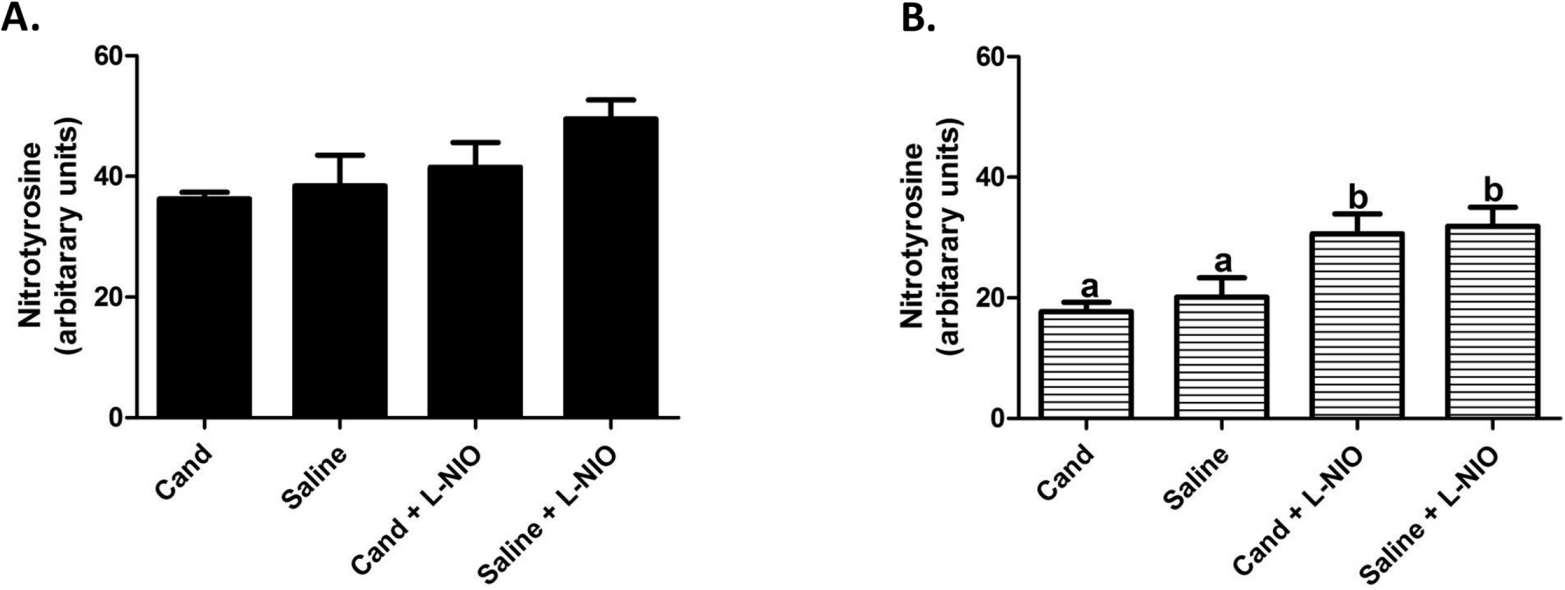
eNOS inhibition alters nitrosative stress levels after stroke. Acute L-NIO treatment did not affect the nitrosative stress levels in the ipsilateral hemisphere ()(A). In contrast, L-NIO induced an increased nitrosative stress in the contralateral hemispheres of both candesartan and saline treated animals (F = 4.76, df = 3;p = 0.008)(B). a, b Pairs of means with different letters are significantly different from each other. Data presented as mean±SEM; n = 6–8 per group.

Our findings demonstrated that eNOS inhibition upregulates nNOS expression after stroke. eNOS inhibition significantly increased the levels of nitrosative stress in the contralesional hemisphere of both candesartan and saline treated animals (Fig 6B). In contrast, eNOS inhibition did not affect the levels of nitrosative stress in the ipsilateral hemisphere (Fig 6A).

### Vascular density was unchanged at 7 days after stroke and treatment

Despite increased growth factor expression in the hypertensive animals of this study, we were unable to detect an effect of treatment on 7 day vascular density in the peri-infarct region (S1 Fig), as we have reported in normotensive animals [3].

## Discussion

Candesartan is an often-prescribed AT1 blocker with robust neurovascular protective properties in experimental stroke [1, 2, 4, 5, 7, 9, 11]. Therapeutic manipulation of the renin-angiotensin system (RAS) for treatment of brain injury remains very promising, and exploration of the molecular pathways involved continue to reveal a broad range of protective and restorative mediators that may be harnessed. The results of this investigation demonstrate the importance of eNOS activation as a unifying mechanism explaining many of the proposed protection pathways. In addition, we demonstrated a vital physiologic role of eNOS activity after stroke in hypertensive animals. Interestingly, our data identified, for the first time, the ability of AT1 blockers to attenuate ischemia-induced ER stress to improve stroke outcome in hypertensive animals. This process was also mediated by the activity of eNOS.

We are not the first to implicate eNOS as a critical factor in neuroprotection after stroke. Experimental data from eNOS knockout animals showed a larger infarct size and blunted ischemia-induced upregulation of growth factors [19]. Despite the well-established effect of chronic eNOS inhibition on stroke outcome [16], the effect of acute inhibition of eNOS on stroke outcome in hypertensive animals and the involvement of eNOS in candesartan-induced neuroprotection and growth factor expression remained undetermined. BDNF has been shown to reduce infarct size and improve functional outcome following cerebral ischemia [22]. Recently, we reported on the ability of candesartan to increase BDNF expression in SHR [27]. Kishi T et al. have also reported the ability of telmisartan, another AT1 blocker, to ameliorate cognitive dysfunction in stroke-prone SHR through the BDNF/TrkB system [29].

Our recent data confirm and further extend data on candesartan’s induction of the BDNF/TrkB system [30]. This effect of ARBs was attributed to unopposed stimulation of AT2 receptor in SHRs as have reported previously [31] [27]. Interestingly, we found that candesartan robustly upregulated mature BDNF in the contralesional hemisphere which has been shown to be involved in recovery through induction of neuroplasticity [32]. This effect is similar to that reported in normotensive animals treated with subhypotensive doses of candesartan [17].

BDNF is widely expressed in various tissues and cell types [21, 33]. Following stroke, the temporal and spatial expression of BDNF has been studied [34]. Endothelial cells have been demonstrated to be a prominent source of BDNF even when compared to neurons [20]. Additionally, we have previously demonstrated the ability of candesartan to upregulate BDNF expression and release in human brain microvascular endothelial cells[27]. Accordingly, in this investigation, the focus was the ability of candesartan to upregulate BDNF expression in hypertensive animals after stroke and to assess eNOS involvement in this proposed effect.

A unique finding in this work is the possible involvement of eNOS in neurotrophin processing in addition to already reported effects on expression [35]. In both candesartan and saline treated animals, eNOS inhibition resulted in an increase in proBDNF levels in both hemispheres. This finding suggests a possible regulatory role of eNOS in processing of proBDNF to mature BDNF. Additionally, proBDNF accumulation in the ipsilateral hemisphere would adversely affect neuronal survival and neuroplastic changes in the penumbra. This finding identifies a possible mechanism through which eNOS inhibition worsens stroke outcome.

Additionally, candesartan increased the levels of mature BDNF while simultaneously reducing the levels of proBDNF in both hemispheres. This finding suggests that candesartan induced increased levels of mature BDNF in the brain is mediated through enhancing proBDNF conversion into the mature form. Recently, we reported a similar pattern in normotensive animals treated with subhypotensive doses of candesartan [17]. Furthermore, we previously reported the ability of candesartan to increase the expression of growth factors at the mRNA level [3]. Guan et al. reported that candesartan induced a minimal but significant increase in the levels of BDNF mRNA. Accordingly, the effect of candesartan on the levels of mature BDNF is mediated primarily through enhancing the conversion of proBDNF. When these two findings are taken together, our findings demonstrate for the first time that the effect of ARBs on the levels of mature BDNF is mediated through enhancing proBDNF conversion into mature BDNF in an eNOS dependent manner.

Another interesting finding of this study was the ability of early AT1 blockade to prevent ischemia induced p75NTR expression. This effect was observed in the ipsilateral hemisphere and was not affected by eNOS inhibition. This finding suggest that candesartan promotes the prosurvival effects of the neurotrophin family while reducing the pro-apoptotic effect of the same family by reducing both p75NTR expression and its ligand proBDNF.

ER stress has been shown to play a role in the pathophysiology of stroke [36] and its alleviation was associated with an ameliorated ischemic insult in diabetic animals [37]. Recently, an association between ER stress and hypertension has been suggested [28]. Additionally, BDNF has been demonstrated to reduce ER stress in neurons and after stroke [38, 39]. Our results showed a robust increased expression of ER stress markers in the ipsilateral hemisphere of hypertensive animals after ischemia induction. Expression of ER stress markers in the contralateral hemisphere was very low. Accordingly, analysis was limited to the ipsilateral hemisphere. To our knowledge, we are the first group to report the ability of AT1 blockers to alleviate ischemia-induced ER stress. This amelioration was demonstrated to be eNOS-mediated and is consistent with BDNF expression after stroke. It is possible that candesartan-induced BDNF expression mediated this anti-ER stress effect.

Previous reports on ER stress in stroke did not assess the cleavage of ATF6 that leads to its nuclear localization and induction of its effects. In this work, we assessed ATF6 cleavage using the same method that has been used recently by Dromparis et al [40]. These findings identify a novel mechanism by which AT1 blockers improve stroke outcome. In addition, it paves the way for further investigation to assess the possible implications of this finding in other disease states where ER stress has been shown to be involved in the pathophysiology.

Reciprocal regulation of different NOS isoforms has been reported previously [41]. Our data support this concept and identify the regulation of nNOS expression as another mechanism by which eNOS activity affects stroke outcome in hypertensive animals. nNOS expression has been shown to worsen stroke outcome by inhibiting neurogenesis [42]. The exact mechanism of nNOS induced neurogenesis inhibition is still unknown. Our results demonstrated an almost three fold increase in Nogo-A expression after eNOS inhibition. This similar expression pattern between nNOS and NOGO-A in response to eNOS inhibition suggests a possible mechanistic link between the two proteins. nNOS activation might inhibit eNOS activity, which we demonstrated to upregulate NOGO-A expression and NOGO-A is known to induce neuronal apoptosis [43]. The concomitant increase in JNK phosphorylation supports this possible link although definitive confirmation requires further mechanistic investigation.

Our findings showed a significant increase in nitrosative stress in the contralateral hemisphere in animals that received LNIO. This finding might reflect the increased production of NO due to L-NIO induced nNOS production. Increased oxidative /nitrosative stress has been shown to inhibit angiogenesis in human retinal cells [44] and is involved in diabetes induced dysfunctional angiogenesis and poor outcome after stroke [45] [46–48]. The contralateral hemisphere is thought to be involved in recovery after stroke [17, 49]. Accordingly, our data provides an intriguing new target to assess the role of eNOS inhibition induced nNOS upregulation in the contralateral hemisphere and its resulting oxidative/nitrosative stress on the functional outcome after cerebral ischemia.

In this investigation, we considered the involvement of different distinct, but closely interconnected pathways in stroke outcome. Despite all efforts made, the following limitations should be highlighted. We used a pharmacologic approach to inhibit eNOS. Although not selective for eNOS, L-NIO is widely used as an eNOS inhibitor [50, 51]. In addition, our interest was to assess the role of acute inhibition of eNOS in hypertensive animals rather than the chronic effects. The rationale behind this preference is based on reports of higher eNOS expression in response to increased oxidative stress and endothelial dysfunction, both of which are considered major pathophysiologic mechanisms in hypertension-induced vascular complications [35]. Accordingly, one of our aims was to elucidate the functional role of acutely increased eNOS expression, caused by candesartan treatment after stroke, in hypertensive animals.

**Fig 7:**
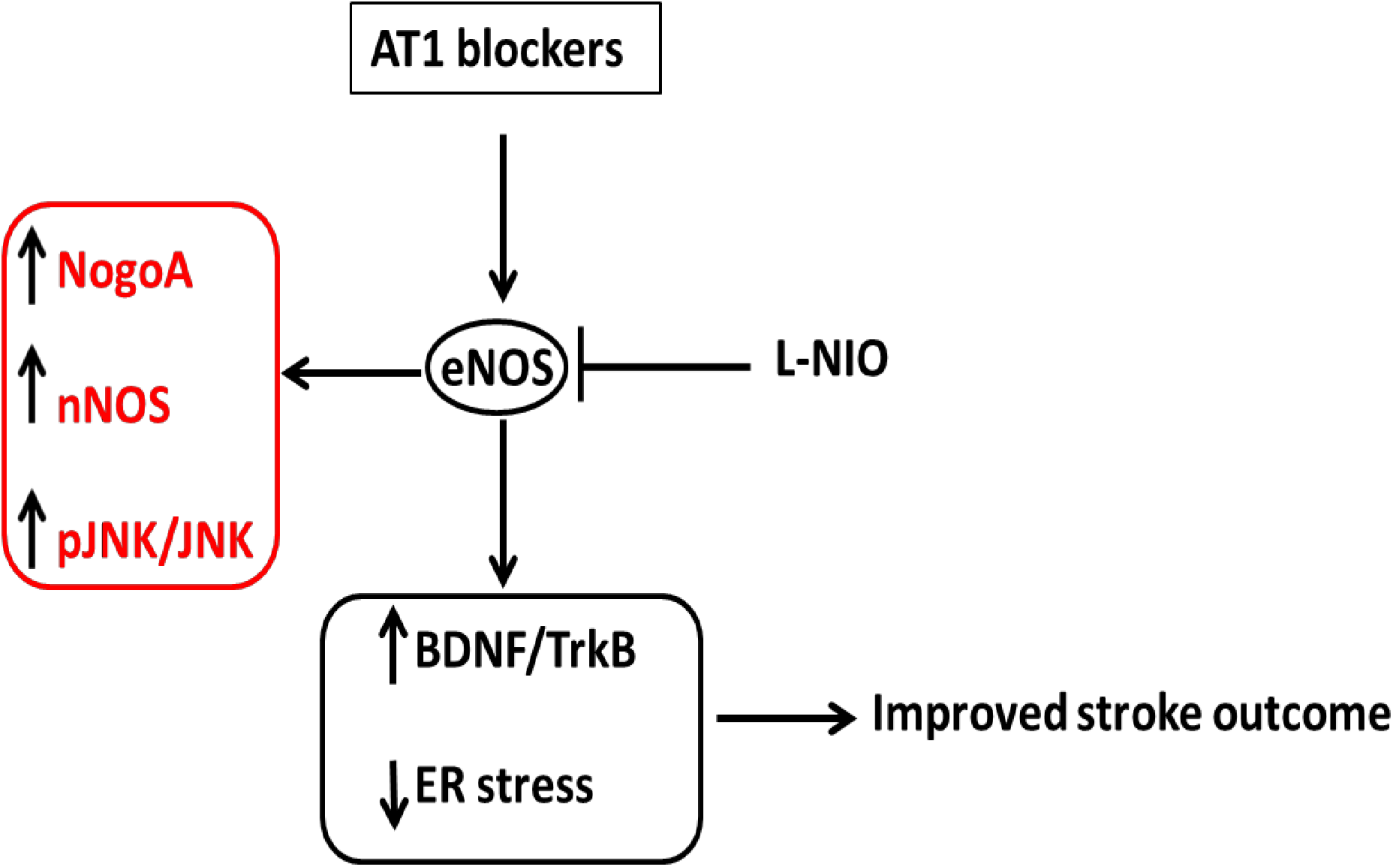
A schematic representation of the main findings. Acute administration of ARBs improves short term functional outcome through an eNOS mediated amelioration of ER stress and BDNF/TrkB mediated signaling. Acute eNOS inhibition increases NOGO-A, nNOS expression and JNK phosphorylation.

In conclusion, our findings demonstrated the ability of candesartan to confer protection after stroke and increase mature BDNF expression in both hemispheres of hypertensive animals in an eNOS-dependent manner. In addition, for the first time, our findings clearly demonstrated that the effect of candesartan on the levels of mature BDNF is primarily mediated through conversion of proBDNF into the mature form. We also demonstrated for the first time the involvement of eNOS in neurotrophin processing in addition to the already established role in neurotrophin expression (Fig 7). In addition, for the first time, our findings demonstrated candesartan’s ability to counteract ischemia-induced ER stress through an eNOS mediated mechanism that might involve BDNF (Fig 7). Finally, our data suggests novel cross talk between NOS isoforms and Nogo-A expression and signaling in hypertensive animals (Fig 7). Since all of these pro-recovery molecular events depend on the presence of eNOS, it is not surprising that the benefits are lost at 7 days after a single dose of candesartan. Unlike their normotensive counterparts, the hypertensive animals in this study likely would require sustained dosing of the ARB after stroke to promote endothelial function and enhance long-term stroke outcome.
